# Methylation of Promoter Regions of Genes of the Human Intrauterine Renin Angiotensin System and Their Expression

**DOI:** 10.1155/2015/459818

**Published:** 2015-03-30

**Authors:** Shane D. Sykes, Carolyn Mitchell, Kirsty G. Pringle, Yu Wang, Tamas Zakar, Eugenie R. Lumbers

**Affiliations:** ^1^School of Biomedical Sciences and Pharmacy, University of Newcastle, Newcastle, NSW 2308, Australia; ^2^Mothers and Babies Research Centre, Hunter Medical Research Institute, Newcastle, NSW 2308, Australia; ^3^School of Medicine and Public Health, University of Newcastle, Newcastle, NSW 2308, Australia; ^4^Department of Obstetrics and Gynaecology, John Hunter Hospital, Newcastle, NSW 2308, Australia

## Abstract

The intrauterine renin angiotensin system (RAS) is implicated in placentation and labour onset. Here we investigate whether promoter methylation of RAS genes changes with gestation or labour and if it affects gene expression. Early gestation amnion and placenta were studied, as were term amnion, decidua, and placenta collected before labour (at elective caesarean section) or after spontaneous labour and delivery. The expression and degree of methylation of the prorenin receptor (*ATP6AP2*), angiotensin converting enzyme (*ACE*), angiotensin II type 1 receptor (*AGTR1*), and two proteases that can activate prorenin (kallikrein, *KLK1*, and cathepsin D, *CTSD*) were measured by qPCR and a DNA methylation array. There was no effect of gestation or labour on the methylation of RAS genes and *CTSD*. Amnion and decidua displayed strong correlations between the percent hypermethylation of RAS genes and *CTSD*, suggestive of global methylation. There were no correlations between the degree of methylation and mRNA abundance of any genes studied. *KLK1* was the most methylated gene and the proportion of hypermethylated *KLK1* alleles was lower in placenta than decidua. The presence of intermediate methylated alleles of *KLK1* in early gestation placenta and in amnion after labour suggests that *KLK1* methylation is uniquely dynamic in these tissues.

## 1. Introduction

Human intrauterine tissues (amnion, chorion, decidua, placenta, and myometrium) all express genes that affect the rate of production and actions of angiotensin peptides [1, 2]. Prorenin (the inactive form of renin) is secreted mainly by decidua. It requires proteolysis or binding to the prorenin receptor (*ATP6AP2*) to become active [3].

There are two potential pathways via which the decidual and amniotic renin angiotensin systems (RASs) could be involved in parturition. First, during labour, there is a marked up regulation of inflammatory genes in term amnion, decidua, and placenta, the so-called “inflammatory signature” [4]. Angiotensin II (Ang II), the major product of the renin angiotensin cascade formed from Ang I by angiotensin converting enzyme (*ACE*) and acting through the angiotensin II type 1 receptor (*AGTR1*), is a proinflammatory peptide [5]. Second, renin and prorenin acting independent of Ang II stimulate prostaglandin endoperoxide synthase-2 (PGHS-2) production in primary amnion cells* in vitro* [6]. Both kallikrein and cathepsin D can proteolytically activate prorenin and are present in human intrauterine tissues [7–10]. Cathepsin D is a lysosomal enzyme that can also form angiotensin I from angiotensinogen. It has been implicated in promoting invasion by endometriotic tissue [8]. Kallikrein, a serine protease, is the key enzyme involved in activation of the kallikrein-kinin system (KKS). The KKS has actions that generally oppose those of the RAS in regulating blood pressure; however, in some circumstances the KKS has effects that are similar to the RAS in regulating vascular function, inflammation, and cell growth [11].

Cell specific DNA methylation contributes to cell identity. Although CpG methylation status is conferred early in development [12–14], it can change [15–17] and could be one pathway regulating intrauterine gene expression throughout gestation. Methylation of CpG dinucleotides in the promoter region of a gene represses transcription either by inhibiting binding of transcription factors or by binding proteins that condense the chromatin structure. Three RAS genes essential for the generation or activity of Ang II in human intrauterine tissues, namely, the prorenin receptor (*ATP6AP2*), angiotensin converting enzyme (*ACE*), and the angiotensin II type 1 receptor (*AGTR1*) have a high density of CpG motifs in their promoter region, as do kallikrein (*KLK1*) and cathepsin D (*CTSD*). We have previously demonstrated that placental expression of* ATP6AP2* and* AGTR1* were highest in early gestation while the expression of* ACE* was greatest at term [1]. We attributed this difference in* ACE* mRNA abundance to the increased density of fetal endothelial cells which are the only placental cells containing ACE [1]. We postulated that if the methylation status of* ATP6AP2* and* AGTR1* influenced their expression then the level of methylation of these two genes would also increase throughout gestation.

The methylation of RAS genes within the intrauterine tissues could also be altered during labour and affect their expression, since decidual* ATP6AP2* and amnion* ACE* expressions are both downregulated in labour [2, 18]. We therefore determined the methylation status of* ATP6AP2*,* ACE*, and* AGTR1* in these tissues across gestation and during labour and correlated this with changes in their expression. In addition, we explored methylation-dependent regulation further by determining for the first time the effects of gestation and labour on expression of* KLKI* and* CTSD* in gestational tissues and correlating expression levels with the methylation density of the respective gene promoters.

## 2. Methods

### 2.1. Ethics Approval

The Hunter Area Research Ethics Committee and the University of Newcastle Research Ethics Committee have given approval to carry out this work.

### 2.2. Tissues

Early gestation placenta (*n* = 4) and amnion (*n* = 8) were collected after informed consent from women undergoing elective termination of pregnancy (10–18 weeks of gestation). Term tissues (37–41 weeks of gestation) were collected from women presenting to the John Hunter Hospital (Newcastle, Australia) with uncomplicated singleton pregnancies following informed consent. Term amnion (*n* = 8), decidua (*n* = 8), and placenta (*n* = 4) were obtained after elective caesarean section in the absence of labour. Amnion and decidua were also collected from women after spontaneous labour and vaginal delivery and processed within 30 min of collection (*n* = 8).

Women were excluded if there was a history of treatment with nonsteroidal anti-inflammatory drugs, infection, chorioamnionitis, asthma, or induced labour. Amnion and decidua were collected from the reflected membranes and dissected at least 1 cm away from the adjacent placenta; amnion was then subsequently peeled from the chorio-decidua and the decidua isolated from the chorion laevae by sharp dissection as described previously [19]. Placental tissue was excised from several sites across the placental bed. Samples used in this study were from a tissue bank of previously collected tissues and thus were not matched by patients. All tissues were snap-frozen in liquid nitrogen for storage at −80°C until further processing.

### 2.3. Methylation Density Analyses

All samples were analysed using a customized Methyl-Profiler DNA Methylation PCR Array System (SA Biosciences, Doncaster, Vic, Australia, distributed by Qiagen as EpiTect Methyl qPCR System) according to the manufacturer's specifications. Briefly, genomic DNA was isolated from 20–25 mg of crushed frozen placenta, amnion, or decidua using the QIAamp DNA mini kit (Qiagen, Chadstone, Vic, Australia) according to the manufacturer's instructions. Genomic DNA was then treated with one of 4 enzyme treatments provided in the DNA Methylation Enzyme Kit (SA Biosciences). Restriction digests using either the methylation-dependent or the methylation-sensitive enzyme contained 1 *μ*L of enzyme solution, while digests containing both enzymes contained 1 *μ*L of each. Mock digests contained no restriction enzyme. Samples were made to a final volume of 30 *μ*L containing 125 ng of isolated genomic DNA in digestion buffer. The four digested DNA solutions were processed for PCR analysis by adding RT^2^ SYBR Green qPCR Master Mix (SA Biosciences) and water to a final concentration of 7.8 ng of DNA per well. The PCR plates were supplied with the appropriate primers for* ACE*,* AGTR1*,* ATP6AP2*,* CTSD*, and* KLK1* genes coated to the wells of each plate. The PCRs were performed on an Applied Biosystems 7500 Real-Time PCR System (Applied Biosystems, Mulgrave, Vic, Australia) according to the manufacturer's instructions. The primer sequences and amplicon positions have been optimized by the manufacturer and are considered proprietary. All amplicons included the transcription start site (TSS), several hundred base pairs upstream and downstream of the TSS, and at least one CpG island including or near to the TSS. The methylation status of the genes was calculated using software supplied with the DNA Methyl-Profiler PCR Array (SA Biosciences) and according to the manufacturer's recommendations. This software reports the proportions of gene alleles within each DNA sample that are hypermethylated, intermediately methylated, or hypomethylated. This technique relies on the ability of the methylation-dependent restriction enzyme (which cleaves methylated DNA) and methylation-sensitive restriction enzyme (which cleaves only at unmethylated recognition sites) to digest the DNA differentially. Assays were reported as failures if delta-Ct values between digests with both restriction enzymes or the mock-digest with neither enzyme were ≤2, representing a sample where ≥25% of the template was digestion-resistant. The principle and validation of this DNA methylation density monitoring system have been published previously [20, 21].

### 2.4. Quantitative Real-Time RT-PCR (qPCR)

Total RNA was isolated from 0.2 g of tissue using TRIzol reagent (Invitrogen, Mulgrave, Vic, Australia) according to the manufacturer's instructions. Samples were purified and contaminating DNA were removed by treatment with DNase I on RNeasy Mini kit spin columns (Qiagen) and quantification and purity of RNA were determined using spectrophotometry (NanoDrop ND-1000, NanoDrop Technologies, Rockland, DE, USA). Reverse transcription was carried out with Superscript III RT-kit using random hexamer primers (Invitrogen). Quantitative real-time PCR was conducted with SYBR Green for detection using an Applied Biosystems 7500 Real-Time PCR System. Each reaction contained 5 *μ*L SYBR Green, cDNA generated from 10 ng of total RNA, primers, and water, made up to a final volume of 10 *μ*L. Primers for* KLK1* were designed as previously described [22], while all other primers except* CTSD* have been described elsewhere [2, 18]. The primers for* CTSD* were designed with Primer Express 3.0 software (Applied Biosystems) using NCBI reference sequence NM_001909.3. The sequences of the forward and reverse primers for detecting* CTSD* mRNA were 5′CCTGAGCAGGGACCCAGAT and 5′GGTGACATTCAGGTAGGACAGAGA, respectively. This set of primers was used at the optimized concentration of 100 nM. All samples were assayed in triplicate using the following reaction conditions: incubation at 50°C for 2 minutes, incubation at 95°C for 10 minutes, 40 cycles of 15 seconds at 95°C, and 40 cycles of 1 minute at 60°C. Dissociation curves were established after all qPCR amplifications to ascertain the homogeneity of the products. Data from qPCR were analysed according to the 2^−ΔΔCt^ method using *β*-actin mRNA as an internal reference and efficiency ratios were verified to be 2 for all genes [23]. The abundance of *β*-actin mRNA was determined to be equal in all gestational and tissue groups except for amnion, in which *β*-actin mRNA levels were 14% less in early gestation than those of the other amnion groups. Therefore, the relative mRNA abundance measured in early gestation amnion samples was multiplied by 1.14 in order to compensate for this difference in the average reference mRNA level. Term placental cDNA was used as a calibrator for all genes except* KLK1*, where cDNA from SW48 cells (a colorectal cancer cell line) was used as the calibrator instead.

### 2.5. Statistics and Data Analysis

Data were analysed using Mann-Whitney *U* tests, Kruskal-Wallis tests with post hoc Dunn's multiple comparison tests, and Spearman correlations. Significance was accepted at the 5% level. Data are presented as Tukey box plots with outliers shown as filled in circles. To determine whether or not any individual gene had a different degree of hypermethylation of alleles from levels measured in the 5 genes measured in this study and the 7 genes studied by [24], significant relationships identified using Spearman correlations are visually represented by scatter plots with logarithmic scales. GraphPad Prism 6.0 (GraphPad Software Inc.) was used for statistical analyses and preparation of figures.

## 3. Results

### 3.1. The Effects of Gestational Age and Labour on RAS,* CTSD*, and* KLK1* Gene Methylation and mRNA Expression in Amnion

**Figure 1:**
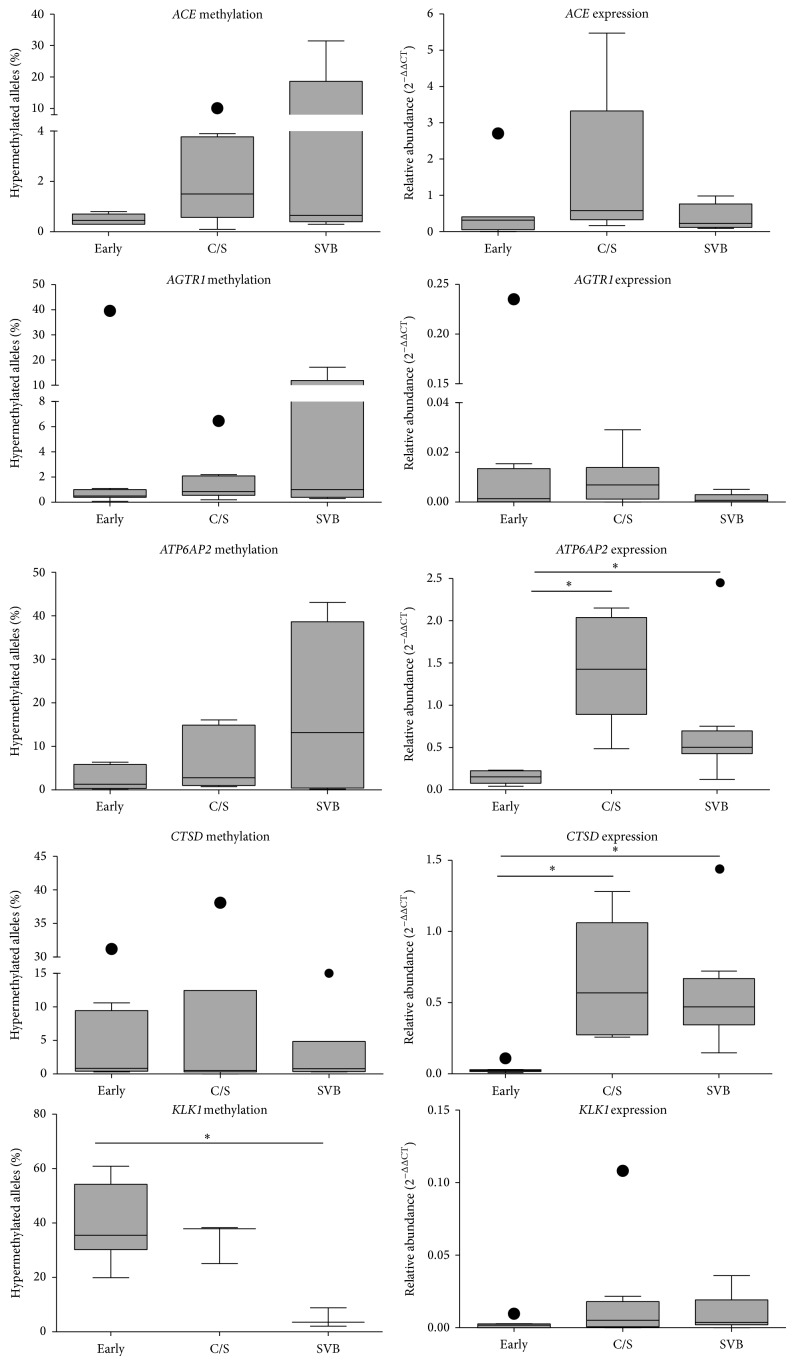
The proportions of hypermethylated gene alleles for* ACE*,* AGTR1*,* ATP6AP2*,* CTSD*, and* KLK1* and their corresponding mRNA abundances in amnion collected from early gestation (early, *n* = 8) and normal term pregnancies either by elective caesarean section prior to the onset of labour (C/S, *n* = 8) or after labour in women who had a spontaneous labour and vaginal delivery (SVB, *n* = 8). Significant differences were determined with Kruskal-Wallis tests using Dunn's multiple comparison test, with significant differences denoted by ∗ (adjusted *P* < 0.05). Data are presented as Tukey box plots with outliers shown as filled in circles. DNA methylation profiles could not be measured in early gestation for 1/8* ACE* and* KLK1* samples, from C/S for 1/8* ATP6AP2*, 2/8* CTSD*, and 5/8* KLK1* samples, and from SVB for 1/8* AGTR1*, 4/8* ATP6AP2*, 2/8* CTSD*, and 5/8* KLK1* samples.

In the amnion, the proportion of hypermethylated* ACE*,* AGTR1*,* ATP6AP2*, and* CTSD* gene alleles varied among individuals but showed no significant differences with gestational age or labour (Figure 1). One early gestation amnion had intermediate methylation in the* ATP6AP2* gene, and there was no intermediate methylation of* ACE, AGTR1*, and* CTSD* genes in any amnion sample. A significantly greater proportion of* KLK1* alleles was hypermethylated in early gestation amnion compared to amnions collected after spontaneous labour and vaginal delivery at term (*P* < 0.02, Figure 1). Furthermore, intermediate* KLK1* methylation density was found in one caesarean derived amnion (hypermethylated, 25.1%; intermediately methylated, 30.6%; and hypomethylated, 44.3%) and 2 amnion samples collected after labour (8.8 and 2.1% hypermethylated; 83.3 and 96.0% intermediately methylated; and 7.9 and 1.9% hypomethylated, resp.).

Amnion* ACE, AGTR1*, and* KLKI* mRNA levels did not change with gestation, but there was a significant increase in* ATP6AP2* and* CTSD* mRNA abundance in late pregnancy after both caesarean and vaginal delivery (for* ATP6AP2*, *P* < 0.001 and *P* < 0.05; for* CTSD*, *P* = 0.001 and *P* < 0.01, resp.). Labour had no effect on the expression of any of these amniotic genes, and we found no correlation between mRNA abundance and the proportion of hypermethylated alleles.

### 3.2. The Effects of Labour on RAS,* CTSD*, and* KLK1* Gene Methylation and mRNA Expression in Decidua

**Figure 2:**
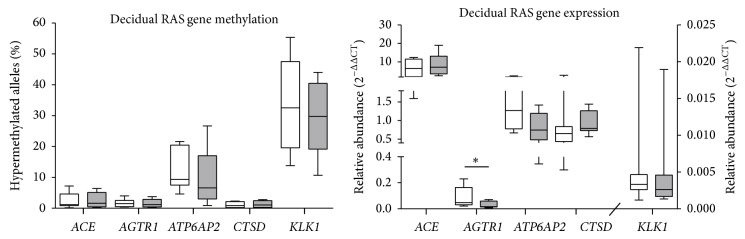
The proportions of hypermethylated alleles of* ACE*,* AGTR1*,* ATP6AP2*,* CTSD*, and* KLK1* and the mRNA abundances of these genes isolated from decidua collected from normal term pregnancies either by elective caesarean section prior to the onset of labour (white bars, *n* = 8) or from women who had spontaneous labour and a vaginal delivery (grey bars, *n* = 8). Significant differences were determined using Mann-Whitney *U* tests, with significant differences denoted by ∗ (*P* < 0.05). Data are presented as Tukey box plots. DNA methylation profiles could not be detected in the absence of labour for 1/8* ACE*, 1/8* AGTR1*, 2/8* ATP6AP2*, and 3/8* KLK1* samples and from after labour for 3/8* KLK1* samples.

There was no effect of labour on the proportion of RAS gene alleles that were hypermethylated (Figure 2) in the decidua. The mRNA abundances of the RAS genes were also unchanged, except for* AGTR1*, which was lower in women who had a spontaneous labour and vaginal delivery than in those who delivered by caesarean section in the absence of labour (Figure 2). In the decidua the mRNA abundances of RAS genes,* CTSD*, and* KLK1* were not correlated with the proportion of hypermethylated alleles.

### 3.3. The Effects of Gestational Age on RAS,* CTSD*, and* KLK1* Gene Methylation and mRNA Expression in Placenta

The proportion of RAS gene alleles that were hypermethylated in the placenta was not affected by gestational age (data not shown). In early gestation placentae, however, several genes showed a high level of intermediate methylation (Table 1). No late gestation placentae showed intermediate methylation; all studied genes were hypomethylated. The small number of samples with intermediate DNA methylation did not allow statistical analyses to be conducted between gestational age groups.

**Figure 3:**
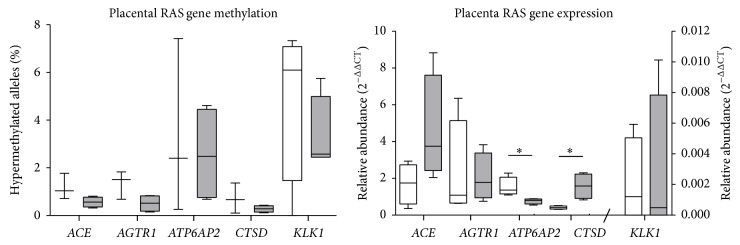
The mRNA abundance of* ACE*,* AGTR1*,* ATP6AP2*,* CTSD*, and* KLK1* in early and late gestation placentae (*n* = 4, resp.). Significant differences were determined using Mann-Whitney *U* tests, with significant differences denoted by ∗ (*P* < 0.05). Data are presented as Tukey box plots.

There were no gestational changes in* ACE*,* AGTR1*, and* KLKI* mRNA levels, but* ATP6AP2* mRNA abundance decreased and* CTSD* mRNA abundance increased with gestational age (Figure 3). We found no correlations in the placenta between mRNA abundance and the proportion of hypermethylated alleles of any gene studied.

### 3.4. Effects of Gestational Tissue Type on the Proportion of Methylated RAS,* CTSD*, and* KLK1* Alleles and mRNA Expression

**Figure 4:**
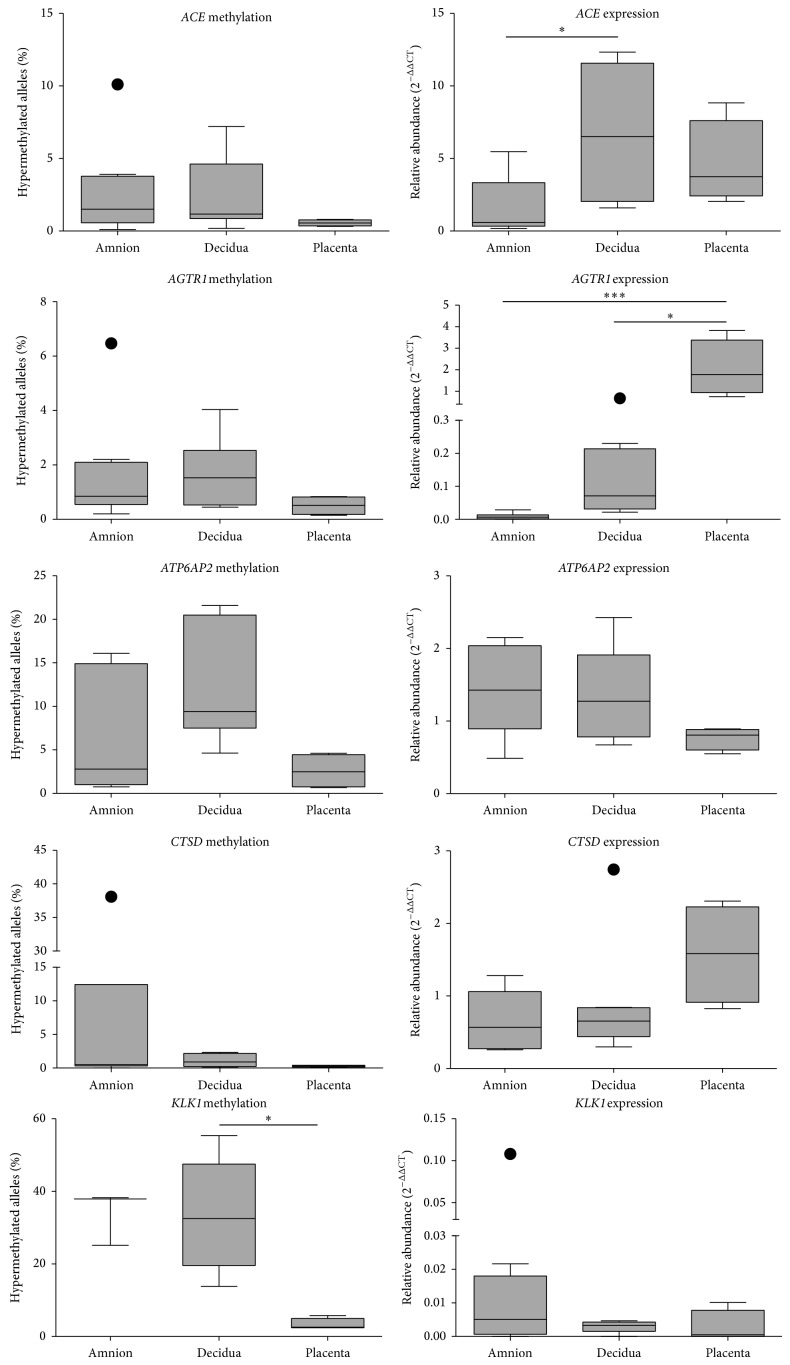
The proportions of hypermethylation in alleles of* ACE*,* AGTR1*,* ATP6AP2*,* CTSD*, and* KLK1* and the mRNA abundance of these genes within amnion, decidua, and placenta collected from term pregnancies before the onset of labour. Significant differences were determined with Kruskal-Wallis tests using Dunn's multiple comparison test, with significant differences to amnion denoted by ∗ (*P* < 0.05) and ∗∗∗ (*P* < 0.001). Data are presented as Tukey box plots with outliers displayed as filled circles.

We compared levels of RAS gene methylation in amnion, decidua, and placenta obtained at elective caesarean section (Figure 4). Only* KLK1* showed tissue specific differential methylation with decidua exhibiting a greater proportion of hypermethylated* KLK1* alleles than placenta (*P* < 0.05). No differences were seen in* KLK1* mRNA abundance between these tissues.

Furthermore,* ACE* mRNA abundance was greater in decidua than in amnion (*P* < 0.05) and placental* AGTR1* mRNA abundance was higher than that measured in both decidua and amnion (*P* < 0.05 and *P* < 0.001, resp.).

### 3.5. Relationships between the Proportions of Methylated RAS,* CTSD*, and* KLK1* Alleles in the Gestational Tissue

**Figure 5:**
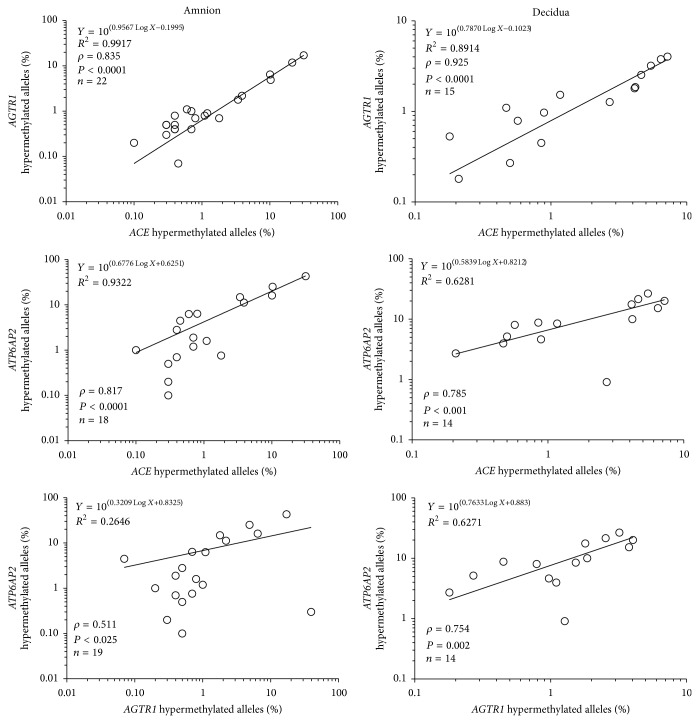
Spearman correlations between the proportions of hypermethylated RAS gene alleles within amnion samples isolated from early and late gestation and within decidua samples collected from before and after the onset of labour.

**Figure 6:**
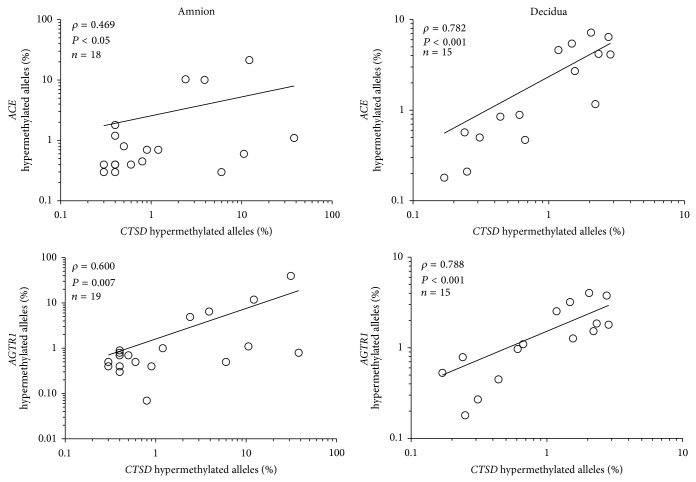
Spearman correlations between the proportion of hypermethylated* CTSD* alleles and the proportion of hypermethylated* ACE* and* AGTR1* alleles in all amnion and decidua samples.* ATP6AP2* was not correlated with* CTSD*.

In amnion and decidua, the proportions of hypermethylated* ACE*,* AGTR1*, and* ATP6AP2* alleles were positively correlated (Figure 5). Furthermore, the proportions of hypermethylated* CTSD* alleles were correlated with the proportions of hypermethylated* ACE* and* AGTR1* alleles (Figure 6) in both tissues. In the placenta, the only correlation found was between the proportions of highly methylated* CTSD* and* KLK1* alleles (*rho* = 0.964, *P* < 0.003, *n* = 7).

To see if the correlated methylation profiles of the 3 RAS genes and* CTSD* in the decidua and amnion were part of a more widespread gene methylation pattern, we extended the correlation analysis to the proportions of hypermethylated labour associated inflammatory gene alleles (*PTGS2*,* BMP2*,* NAMPT*, and* CXCL2*) and steroid receptor gene alleles (*PGR*,* ESR1*, and* NR3C1*) in these tissues [24]. We found that hypermethylation of most of these genes correlated with the hypermethylation of RAS and* CTSD* in the amnion (Table 2). Within the decidua, the hypermethylation of* PTGS2*,* BMP2*,* CXCL2*, and* PGR* also correlated with the RAS genes (Table 2). The proportion of hypermethylated* KLK1* alleles, however, did not correlate with any other gene in either amnion or decidua, including labour associated inflammatory genes and steroid receptor genes.

## 4. Discussion

The principal aim of this project was to explore whether the methylation of RAS and prorenin-activating protease gene promoters influenced the expression of these genes in gestational tissues during pregnancy and labour. We also aimed to assess whether the degree of methylation of these genes were similar to each other, to labour-promoting inflammatory genes, and to genes that encode steroid receptors involved in control of labour [24].

The gestational changes in intrauterine tissue mRNA abundances identified in this study are similar to those that we have previously reported for* ACE*,* AGTR1*, and* ATP6AP2* [1, 2]. The mRNA abundance of* CTSD* has not been determined previously in the human amnion. This study demonstrates that in amnion and placenta, tissues of fetal origin,* CTSD* expression is higher at term. Cathepsin D is a protease involved in trophoblast invasion in the mouse [25, 26] and when activated it can cause apoptosis [27], and protein degradation potentially contributing to fetal membrane remodelling at labour.

*KLK1* expression has previously been detected in very early gestation human placental tissue and decidua by* in situ* hybridization, but it has not been measured in amnion [9, 10]. Apart from activating prorenin, kallikrein can also produce bradykinin, which through the type 2 bradykinin receptor promotes vasodilation, angiogenesis, and invasion in intrauterine tissues [28]. Recently, it has been demonstrated that expression of the type 2 bradykinin receptor is upregulated in decidua and is significantly downregulated in chorionic villous samples from women who develop severe preeclampsia compared to healthy controls [29]. Kallikrein also stimulates the synthesis of labour-promoting PGE in the chorion [30], and drugs that block kallikrein formation delay the onset of labour [31].

There was no relationship in any tissue between the proportions of hypermethylated alleles and the level of expression of the genes. The majority of the alleles were hypomethylated in all tissues, however, indicating that most gene copies were poised for expression under the control of transcriptional regulators.

Early gestation amnion (*n* = 7) had a significantly higher proportion of hypermethylated* KLK1* alleles compared with amnion isolated after spontaneous labour and delivery. At the same time, several term amnion samples showed high proportions of intermediately methylated* KLK1* alleles, which suggests that* KLK1* may be in a state of transition to low methylation density at late gestation. Thus methylation appears dynamic in particular gene(s) even at term, although the impact of this methylation change on* KLK1* gene activity remains unclear.

The wide ranging variation in methylation of RAS genes between individuals, the strong correlations between the proportions of hypermethylated alleles of the 3 RAS genes and* CTSD* in amnion and decidua, and the correlations between methylation profiles of these 4 genes and labour associated gene alleles and steroid receptor gene alleles [32] in amnion suggest that there is a global influence on DNA methylation in the gestational tissues affecting numerous genes. The level of DNA methylation of these genes is set possibly by individual conditions early in gestation [33].

In placentas, high proportions of intermediately methylated alleles were found for several genes (*AGTR1*,* ATP6AP2*,* CTSD*, and* KLK1*) at early gestation (<18 weeks). In contrast, none of the placental samples collected at term in the absence of labour (>37 weeks of gestation) contained detectable levels of intermediately methylated alleles of these genes, however, and the promoters were predominantly hypomethylated. This observation is consistent with the view that DNA methylation is most dynamic in early pregnancy and in the placenta low global methylation levels are established [12].

In conclusion, the promoter methylation status of the studied RAS and renin-activating protease genes has no apparent influence on either the gestational or the labour associated changes of expression in intrauterine tissues reported in previous studies [1, 34]. Promoter methylation is dynamic in the placenta at early pregnancy and in the case of one gene (*KLK1*) in late gestation amnion. The majority of the allele populations are unmethylated, however, and these gene copies may be subject to transcriptional regulation. Future studies focusing on histone modifications may reveal epigenetic mechanisms that have an impact on gene activity in the context of placental function, pregnancy maintenance, and parturition.

## Figures and Tables

**Table 1:** The percentage of gene alleles that were hypermethylated, intermediately methylated, and hypomethylated for *ACE*, *AGTR1*, *ATP6AP2*, *CTSD*, and *KLK1* in placenta from women in early gestation (*n* = 4) or at delivery in late gestation in the absence of labour (*n* = 4, C/S).

Gene	Sample	Early gestation			C/S		
		% Hypermethyl.	% Inter. methyl.	% Hypomethyl.	% Hypermethyl.	% Inter. methyl.	% Hypomethyl.
*ACE *	1	1.0	0.0	99.0	0.3	0.0	99.7
	2	—	—	—	0.8	0.0	99.2
	3	1.8	0.0	98.2	0.5	0.0	99.5
	4	0.7	0.0	99.3	0.7	0.0	99.3

*AGTR1 *	1	1.8	0.0	98.2	0.3	0.0	99.7
	2	—	—	—	0.8	0.0	99.2
	3	1.5	0.0	98.5	0.2	0.0	99.8
	4	0.7	99.3	0.0	0.8	0.0	99.2

*ATP6AP2 *	1	2.4	0.0	97.6	4.6	0.0	95.4
	2	7.4	92.4	0.2	1.0	0.0	99.0
	3	0.3	78.0	21.7	4.0	0.0	96.0
	4	—	—	—	0.7	0.0	99.3

*CTSD *	1	0.7	0.0	99.3	0.1	0.0	99.9
	2	—	—	—	0.3	0.0	99.7
	3	1.4	0.0	98.6	0.4	0.0	99.6
	4	0.1	99.8	0.1	0.2	0.0	99.8

*KLK1 *	1	5.8	0.0	94.2	2.4	0.0	97.6
	2	6.4	93.6	0.0	5.8	0.0	94.2
	3	7.3	0.0	92.7	2.7	0.0	97.3
	4	0.0	100.0	0.0	2.5	0.0	97.5

Inter. = intermediate, methyl. = methylation. Samples with no value (—) had a DNA template in which ≥25% was digestion-resistant from combined methylation sensitive and resistant enzymes.

**Table 2:** Associations between labour associated inflammatory genes (*PTGS2*, *BMP2*, *NAMPT*, and *CXCL2*) and steroid receptor genes (*PGR*, *ESR1*, and *NR3C1*) allele hypermethylation and the percentage of gene alleles that were hypermethylated in *ACE*, *AGTR1*, *ATP6AP2*, *CTSD*, and *KLK1* in amnion and decidua.

	*ACE1 *	*AGTR1 *	*ATP6AP2 *	*CTSD *	*KLK1 *
Amnion					
*PTGS2 *	*r* = 0.85	*r* = 0.81	*r* = 0.50	*r* = 0.60	ns
	*P* < 0.001	*P* < 0.001	*P* = 0.036	*P* < 0.01	
*BMP2 *	*r* = 0.83	*r* = 0.90	*r* = 0.57	*r* = 0.82	ns
	*P* < 0.001	*P* < 0.001	*P* = 0.017	*P* < 0.001	
*NAMPT *	*r* = 0.71	*r* = 0.72	ns	*r* = 0.68	ns
	*P* < 0.001	*P* < 0.001		*P* = 0.001	
*CXCL2 *	*r* = 0.94	*r* = 0.80	*r* = 0.69	*r* = 0.56	ns
	*P* < 0.001	*P* < 0.001	*P* = 0.002	*P* = 0.013	
*NR3C1 *	*r* = 0.72	*r* = 0.78	*r* = 0.49	*r* = 0.49	ns
	*P* < 0.001	*P* < 0.001	*P* = 0.035	*P* = 0.03	
*PGR *	*r* = 0.85	*r* = 0.83	*r* = 0.53	*r* = 0.71	ns
	*P* < 0.001	*P* < 0.001	*P* = 0.02	*P* < 0.001	
*ESR1 *	*r* = 0.87	*r* = 0.77	*r* = 0.71	*r* = 0.49	ns
	*P* < 0.001	*P* < 0.001	*P* = 0.001	*P* = 0.038	

Decidua					
*PTGS2 *	*r* = 0.75	*r* = 0.72	ns	*r* = 0.86	ns
	*P* = 0.001	*P* = 0.003		*P* < 0.001	
*BMP2 *	*r* = 0.90	*r* = 0.90	*r* = 0.62	*r* = 0.84	ns
	*P* < 0.001	*P* < 0.001	*P* = 0.033	*P* < 0.001	
*NAMPT *	*r* = 0.55	ns	ns	*r* = 0.83	ns
	*P* = 0.04			*P* < 0.001	
*CXCL2 *	*r* = 0.88	*r* = 0.82	*r* = 0.78	*r* = 0.77	ns
	*P* < 0.001	*P* < 0.001	*P* = 0.001	*P* = 0.001	
*NR3C1 *	ns	ns	ns	ns	ns
*PGR *	*r* = 0.93	*r* = 0.94	*r* = 0.71	*r* = 0.87	ns
	*P* < 0.001	*P* < 0.001	*P* = 0.005	*P* < 0.001	
*ESR1 *	*r* = 0.72	*r* = 0.80	ns	*r* = 0.83	ns
	*P* = 0.003	*P* < 0.001		*P* < 0.001	

ns = not significant.
